# On the Relation between Assistive Technology System Elements and Access to Assistive Products Based on 20 Country Surveys

**DOI:** 10.3390/healthcare11091313

**Published:** 2023-05-03

**Authors:** Johan Borg, Mikael Winberg, Arne H. Eide, Irene Calvo, Chapal Khasnabis, Wei Zhang

**Affiliations:** 1 School of Health and Welfare, Dalarna University, 79131 Falun, Sweden; 2 Department of Science and Mathematics Education, Umeå University, 90187 Umeå, Sweden; mikael.winberg@umu.se; 3 Department of Health Research, SINTEF Digital, 0373 Oslo, Norway; arne.h.eide@sintef.no; 4 Assistive Technology Access Team, Health Product Policy and Standards Department, World Health Organization, 1211 Geneva, Switzerland; calvoi@who.int (I.C.); chapalk@outlook.com (C.K.); wzhang@who.int (W.Z.)

**Keywords:** access, assistive products, assistive technology, systems

## Abstract

The objective of this study was to explore the relationship between assistive technology system elements and access to assistive products. Data on assistive technology system elements and self-reported survey data on access to assistive products from 20 countries were analyzed using multivariate statistical methods, including orthogonal partial least squares analyses. Access to assistive products was primarily associated with the geographic coverage of assistive technology services in a country, followed by system elements related to policy and personnel. To achieve universal access to assistive technology, geographic coverage of assistive technology services is an instrumental system element. However, it requires the implementation of appropriate policies along with sufficient funding, recruitment of adequately trained personnel, and availability of assistive products in need.

## 1. Introduction

Given the limited access to assistive products across the globe, the World Health Assembly adopted resolution WHA 71.8 on improving access to assistive technology, urging countries to develop, implement, and strengthen policies, programs, and other measures within universal health and social services coverage [1]. This has prompted countries as well as organizations, academia and the industry to accelerate their efforts to address this situation at national, regional and global levels [2,3,4]. For example, to support countries and other stakeholders in this endeavor, the World Health Organization (WHO) has developed tools for assessing a country’s capacity to finance, regulate, procure and provide assistive technology, and for measuring the need for, use of and experiences of assistive technology [5,6].

Assistive technology is the application of organized knowledge and skills related to assistive products, including systems and services [7]. Assistive products are any external products, specially produced or generally available, the primary purpose of which is to maintain or improve functioning and independence, and thereby promote well-being. They can also be used to prevent impairments and secondary health conditions [7]. Examples of assistive products include reminder apps, communication boards, incontinence pads, hearing aids, wheelchairs, and text-to-speech systems. Assistive technology plays a central role in the realization of the Convention on the Rights of Persons with Disabilities [8] by promoting participation and inclusion in society, as well as supporting access to health, education, work, and other important life experiences [9]. Moreover, assistive products are instrumental in achieving all sustainable development goals, which emphasizes the importance of people in need being able to access them [10].

To provide a structure for the assistive technology system, and thereby enable a systematic approach to improving it, the WHO has developed the 5P people-centered assistive technology model. It consists of five components: People, Products, Provision, Personnel and Policy [7], and is an adaptation of the six building blocks of the WHO health systems framework [11]. Strengthening the assistive technology system would mean addressing key constraints in each of the five system components of the 5P model [11]. Therefore, knowing which assistive technology system elements are vital in order to improve access would help guide the planning of and investments in assistive technology programs. Moreover, although the 5P model has been used deductively to categorize elements of the assistive technology system into components, attempts to empirically assess the relevance of its five components have not yet been reported.

Being an integral part of universal health coverage, universal access to assistive technology is a state where everyone, everywhere receives the assistive technology they need without financial or other hardships [12]. However, the knowledge about needs and access has been limited as country-level population data on levels of access to assistive technology have been scarce [13,14]. A recent scoping review among European countries highlighted that data on assistive technology use and need are limited and that comprehensive and disaggregated data is necessary for the development of relevant policies and action. Comparison of data is often problematic due to differences in data collection strategies [15]. To address this, and better understand the global access situation, the WHO launched a multi-country study in 2021, supporting countries and other actors in collecting self-reported population data on access to assistive products [16]. It was found that access, defined as the ratio of the prevalence of met need for assistive products to the prevalence of the sum of met and unmet need for assistive products, varied considerably across the world, from less than 3% in some countries to 90% in others [7].

To complement the multi-country study of access, the WHO undertook a government survey in 2021 to map the assistive technology system-preparedness in countries across a range of system elements, that is, areas mentioned in the resolution on improving access to assistive technology [17]. These system elements can be mapped to the components of the 5P model. The survey found that 69 (99%) of the 70 responding countries had at least one ministry or authority responsible for access to assistive technology, 63 (90%) had at least one measure in place to cover user costs, 56 (80%) had at least one public budget allocated for assistive technology, 62 (89%) had at least one piece of legislation on access to assistive technology, and 53 (76%) had at least one regulation, standard or protocol on assistive technology or accessibility in place. Moreover, 21 (30%) of the responding countries reported having assistive technology services in place for all functional domains (cognition, communication, hearing, mobility, self-care, and vision) across their entire territory while 34 countries (49%) reported that services were available only for some functional domains, or only in some geographical areas. Only seven countries (10%) reported adequate and trained human resources at all levels for all functional domains [7]. This can be compared with the findings of a survey on government action on the implementation of the UN Standard Rules on the Equalization of Opportunities for Persons with Disabilities in 2004–2005 [18]. It reported that 91% of 114 responding countries were involved in the provision of assistive technology, 64% paid or allocated financial resources for the provision of assistive technology, 52% had assistive technology-related policies in place, and 50% had passed related national legislation.

To our knowledge, there has not been any previous international study of the relationship between the characteristics of different assistive technology systems and population-level access to assistive products. However, to inform the implementation of the WHA resolution on improving access to assistive technology, there is a need for evidence supporting the development of strategies that effectively contribute to improving access to assistive products. Therefore, the objective of this study was to explore the relation between assistive technology system elements and access to assistive products.

## 2. Materials and Methods

This study applied a cross-sectional design using two sets of data.

### 2.1. Government Survey

Based on the requested actions in WHA 71.8, the WHO developed a set of ten indicators to measure the status of Member States’ assistive technology system preparedness in terms of legislation, population coverage, geographic coverage, budget, responsible ministries, human resources, education and training, financial coverage, regulations and standards, and specific assistive technology initiatives [17]. Following the drafting of the questionnaire, the questions were reviewed, revised and tested in several iterations. Reviewing and testing were undertaken by members of the Expert Advisory Group to the WHO and UNICEF Global Report on Assistive Technology [7], WHO regional advisors, policy researchers, and independent experts on assistive technology. A web-based questionnaire in English was developed and used to collect data [19]. A paper-based version of the questionnaire was used for a few countries.

Constituting the sampling frame, all 194 Member States of the World Health Organization were invited to participate in the government survey. It was made available from April to December 2021 to focal persons from ministries of health or other relevant ministries or government agencies. A total of 70 governments (36.1%) from all six WHO regions of the world responded [7].

### 2.2. Population Surveys

The method for collecting data on access to assistive products including and excluding spectacles is described in [7,16]. Nationally and regionally representative surveys using the rapid Assistive Technology Assessment (rATA) tool [6] were carried out in 29 countries. Government surveys had been undertaken in 21 of these countries. One country was excluded as the responses to the government survey lacked consistency, which led to the inclusion of 20 countries (see Supplementary Materials Table S1).

Computer assisted personal interviewing (CAPI) was used in 16 of the included countries (in which all household members were interviewed), computer assisted telephone interviewing (CATI) was used in two countries (in which only one person per household was interviewed), a combination of CAPI and CATI was used in one country, and a combination of computer assisted web interviewing (CAWI) and CATI was used in one country (in which not all members of a household were interviewed). The total sample comprises 224,424 participants, with sample sizes ranging from 1479 to 62,723 in individual countries.

### 2.3. Variables

As the prevalence of use and need for spectacles was high compared to other types of assistive products [7], analyses of both access including spectacles and access excluding spectacles were undertaken in this study.

Each independent variable is a composite measure based on the individual measures in Supplementary Materials Table S2. Among the independent variables, ‘functional domains’ refers to cognition, communication, hearing, mobility, self-care, and vision.

#### 2.3.1. Independent Variables from the Government Survey

- *Sectors with legislation*: Number of government sectors with assistive technology-related legislation (0 to 7)
- *Functional domains covered by legislation*: Number of functional domains covered by assistive technology-related legislation (0 to 6)
- *Sectors with budgets*: Number of government sectors with budgets that cover assistive technology (0 to 7)
- *Responsible ministries*: Number of ministries responsible for assistive technology (0 to 6)
- *Districts with services related to [functional domain]*: The proportion of districts (or similar) with assistive technology services related to the functional domain (0% to 100%)
- *Functional domains with adequate personnel*: Number of functional domains with adequate human resources for assistive technology (0 to 6)
- *Functional domains with training*: Number of functional domains for which training on assistive technology is available (0 to 6)
- *Financial measures*: Number of financial measures related to assistive technology (0 to 5)
- *Regulatory measures*: Number of regulatory measures related to assistive technology (0 to 7)
- *Specific initiatives*: Number of specific assistive technology initiatives (0 to 8)

In Table 1, the independent variables, i.e., the studied assistive technology system elements, are mapped to the components of the 5P model [2,7]:

- People—users of assistive products and their families
- Products—assistive products and their development, production and supply
- Provision—system for service provision, including procurement
- Personnel—staff in all related areas
- Policy—legislation, regulatory frameworks, financing mechanisms, information systems

Details about the studied system elements are provided in Supplementary Materials Table S2.

#### 2.3.2. Dependent Variables from Population Surveys

- *Access including spectacles*: Ratio of the prevalence of met need for assistive products including spectacles to the prevalence of met and unmet need for assistive products including spectacles. (0% to 100%)
- *Access excluding spectacles*: Ratio of the prevalence of met need for assistive products excluding spectacles to the prevalence of met and unmet need for assistive products excluding spectacles. (0% to 100%)

### 2.4. Data Analyses

The multivariate relationships between the independent and dependent variables were analyzed using Orthogonal Partial Least Squares (OPLS) [20] in SIMCA 17.0.2. Separate models were calculated for the two dependent variables (including and excluding spectacles, respectively).

OPLS is an extension of the partial least squares technique (PLS) [21], well suited for multivariate regression analyses in the presence of multicollinearity among independent variables (X), noise in both X and dependent variables (Y), and when the number of variables (*k*) is greater than the number of observations (*n*) (and any other combination of *k* and *n*). As in PLS, the variation in the X data is divided into systematic variation and noise (residuals) and the relationship between the systematic variation in X data and Y data is computed. However, in OPLS, the systematic variation in X is further divided into a variation that is predictive of Y and a variation that is not (i.e., that is orthogonal to Y). This improves model interpretability and improves the detection of outliers, due to the eliminated influence of orthogonal variation on the calculations of Hotelling *T*^2^ or DmodX (described below) statistics [20].

Prior to the analysis, all variables were mean-centered and scaled to unit variance to avoid undue influence on the model based on differences in the scale of measurement. All models were evaluated for significance (i.e., if they significantly reduced the cross-validated residuals compared to the global variation around the mean, CV-ANOVA [22]), and both strong and moderate outliers among the countries (i.e., Hotelling’s *T*^2^ Range and normalized distance to the model, DmodX, respectively). The models’ ability to describe or explain the variation in the Y data (i.e., the level of access), is expressed by the term *R*^2^. Since *R*^2^ generally increases with the number of model components calculated, cross-validation was used to determine the number of components to retain to avoid overfitting the models and the decreased generalizability that follows from this. In the cross-validation procedure, one-seventh of the data was left out and predicted by the remaining data and the process was repeated until all data had been left out and predicted once. The predicted values were then compared to the observed values. For the final model, the squared differences between predicted and observed values were summarized, to form the predictive residual sum of squares (PRESS). The ability of the model to predict the omitted values is expressed as *Q*^2^ = 1 − PRESS/SSY, where SSY represents the total variation in the Y matrix after mean centering and UV-scaling. The *Q*^2^ statistic can also be considered as a measure of the extent to which the pattern found in the data generalizes to data outside the model, while *R*^2^ is a measure of how well the model fits the data it is based on. To be retained, a new component must add significantly to the predictive ability (i.e., *Q*^2^) of the model.

To assess the risk of the OPLS models fitting the data well but predicting new observations well purely by chance, we performed response permutation tests [23] in which 100 parallel models were calculated where the X data were kept intact, but the Y data were randomly permutated. The *R*^2^ and *Q*^2^ of these permutated models were then compared to the original model. If valid, the original model should have higher *Q*^2^ and *R*^2^ than the permutated models. Moreover, when the models’ *Q*^2^:s (*y*-axis) are plotted against the degree of correlation between the original and the permutated Y data (*x*-axis), the intercept should cross the *y*-axis at or below zero [24].

### 2.5. Ethical Approval

For the population surveys, general ethical approval was obtained from WHO Ethics Review Committee and individual ethical approvals were obtained from concerned authorities in each country.

## 3. Results

Table 2 provides country-wise survey characteristics and access levels including and excluding spectacles. Table 3 summarizes data for the independent and dependent variables. Detailed data for the included countries are provided in Supplementary Materials Table S1 and data for the individual measures of the independent variables are in Supplementary Materials Table S2.

No strong outliers were found in any of the models (i.e., Hotelling’s *T*^2^ for the countries did not exceed the critical limit at the 0.05 level). With respect to moderate outliers (DmodX), the residuals of Iraq exceeded the critical distance to the model at the 0.05 level. However, excluding Iraq did not affect the results in any significant way. That is, the relative importance of the independent variables for predicting access remained essentially the same (i.e., the same variables were significant and in an identical order of importance). Thus, Iraq was retained in the final models.

The model of access *including* spectacles was significant according to CV-ANOVA *F*(2, 17) = 6.007, *p* = 0.011. The response permutation test indicated that the model was not spurious (i.e., fitted the data well by chance) as the *Q*^2^ of the original model was significantly higher than for the permutated models *t*(99) = −19.076, *p* < 0.001 and the intercept was -0.44. Additionally, the model of access *excluding* spectacles was significant according to CV-ANOVA, *F*(2, 17) = 3.074, *p* = 0.036, and the response permutation test, with a *Q*^2^ significantly higher for the original model than for the permutated models *t*(99) = −21.647, *p* < 0.001, and an intercept of −0.34. In both models, the independent variables were able to explain a substantial amount of the variation in access, i.e., the extent to which the needs for assistive products were met, see Table 4, where *R*^2^ = 59% including spectacles and *R*^2^ = 51% excluding spectacles.

**Figure 1:**
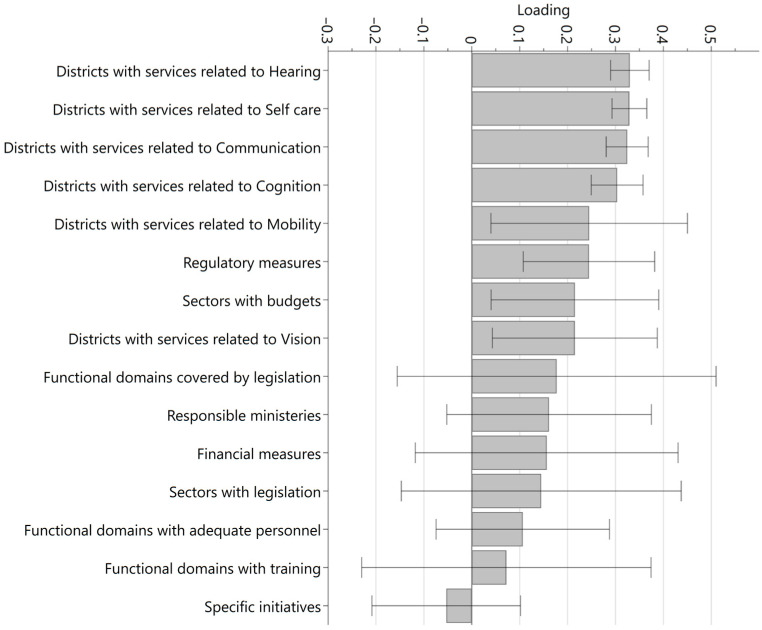
Loading values of variables in the model predicting access including spectacles.

**Figure 2:**
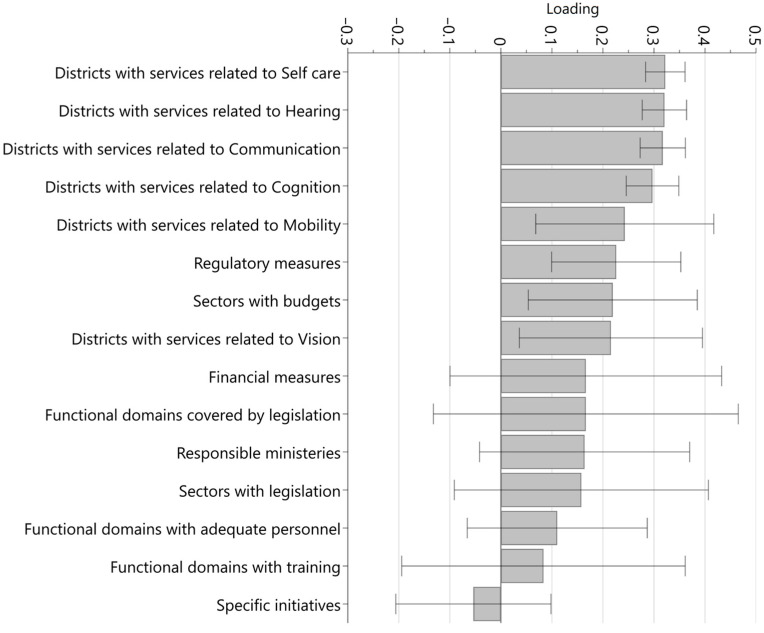
Loading values of variables in the model predicting access without spectacles.

Access *including* spectacles was primarily predicted by the proportion of districts with assistive technology services in the functional domains of hearing, self-care, communication and cognition, in that order (Figure 1). Service coverage related to mobility and vision, respectively, were also significantly and positively related to access, but the point estimates of these variables had considerably larger confidence intervals than for the other assistive technology services. Next to the service coverage, the number of regulatory measures and the number of government sectors with budgets were also significantly and positively associated with access. A positive, but non-significant, association with access was found for the number of functional domains covered by legislation, the number of responsible ministries, the number of government sectors with legislation, the number of functional domains with adequate human resources, and the number of functional domains for which training is available. The association between access and the number of specific assistive technology initiatives was negative, though not significant. A near-identical pattern was found for access *excluding* spectacles (Figure 2).

To further explore the specific initiatives, the analyses were re-run, replacing the Specific initiatives variable with its eight constituent variables (see Supplementary Materials Table S2): *Affordability of assistive products, Development of assistive products, Procurement of assistive products, Service delivery capacity, Collection of data on population-based needs, Information to users and their families, Participation of assistive product users in planning and monitoring services, International collaboration on manufacturing, procurement or supply of assistive products.* The analyses revealed that the association between access and *Collection of data on population-based needs* was slightly positive, while the associations between access and the other initiatives were nearly non-existent or negative. However, none of the associations were statistically significant in predicting access, neither including nor excluding spectacles.

By considering the six variables of districts with services as a single system element and ranking all system elements according to the loading values of the variables, the system elements group themselves according to the 5P components, see Table 5.

## 4. Discussion

### 4.1. Discussion of Findings

Based on self-reported data on access to assistive technology from 20 countries and assistive technology system preparedness data from their governments, this study explored the relationship between assistive technology system elements and access to assistive products. All studied system elements except specific initiatives had a positive association with access to assistive products. The largest statistically significant associations were found for the proportion of districts with services, regulatory measures and sectors with budgets.

It is clear from the results that the assistive technology system elements related to Personnel, Policy and Provision were positively associated with access to assistive products. This means that they need to be considered when developing systems for universal access to assistive technology, supporting previous observations on the importance of each of the elements [7]. Focusing on developing or strengthening a single element or component of an assistive technology system in a country is likely not optimal unless the other elements or components are already sufficiently developed or strengthened.

Previous studies have stressed the importance of providing assistive products close to where people live as distance and traveling costs are significant barriers [25,26], which supports the finding that access is associated with geographic coverage of services. A recommendation to develop an international standard for assistive technology provision to assuring the availability and accessibility of assistive technology addressed several of the elements in this study, including the availability of assistive products, information systems, involvement of both professionals and users, eligibility, funding mechanisms, maintenance and repair, and the service delivery process [27]. To this list, the findings of this study strongly suggest that geographic coverage of services be explicitly added to achieve universal access to assistive technology.

**Figure 3:**
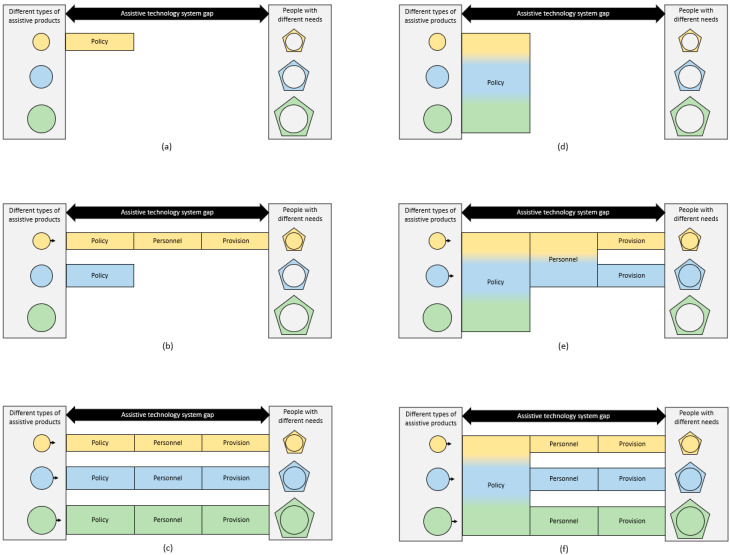
Ways to bridge the assistive technology system gap. Having only one system component for one type of assistive product in place to bridge the gap between available assistive products and people in need does not lead to access, see (**a**). Having all system components in place for one type of assistive product leads to access to that assistive product, see (**b**). Having all system components in place for all types of assistive products leads to access to all assistive products, see (**c**). Having one combined system component for all types of assistive products does not lead to access, see (**d**). Having combined and separate system components in place leads to access to those assistive products for which all system components are in place, see (**e**,**f**).

Figure 3 illustrates the importance of having all components of the 5P model in place to bring Products to People across the access gap using Personnel, Policy and Provision as a bridge. If one component is weak or missing, universal access to assistive technology will not be achieved. For example, from the perspective of access, there is no point in having safe and effective products without sufficient provision or having a comprehensive policy without adequate personnel. However, when developing a system, these components may support each other sequentially or simultaneously, for example, having a policy in place will support the development of the personnel and provision components, and having some trained personnel will likely support the development of both policy and provision which in turn will further support the development of personnel.

Where resources are limited, it is likely better to begin by developing a system with all components in place for a limited range of assistive products, and then step by step broaden each component in a synchronous fashion until universal access to all the assistive technology a population needs is achieved. Stressing the importance of progressive realization, MacLachlan and Scherer state that “embracing the complexity and inter-relatedness of a problem may make it seem insurmountable; and so smaller incremental wins may be targeted over more fundamental systems change” [28] (p.495). The Global Report on Assistive Technology acknowledges this as well and provides recommendations to progressively develop and strengthen assistive technology systems [7]. In this process, each country ought to consider its own context and resources when planning and developing the best path to improve access [7]. Moreover, supporting countries’ efforts to improve access to assistive technology should be an integral part of international collaboration [8].

The findings of this study indicate that specific assistive technology initiatives (such as initiatives focusing on product development, affordability, procurement, information, participation of users in planning and monitoring, and international collaboration) do not necessarily contribute to improving access to assistive products. There may be other impetuses for some of these initiatives, for example, the Convention on the Rights of Persons with Disabilities [8]. However, to achieve universal access, the findings reveal that it is better to develop or strengthen the main system elements or components rather than focussing on isolated initiatives.

The findings lend empirical support to the 5P model and its components as described in the Global Report on Assistive Technology [7]. They also support its breadth of recommendations and actions while cautioning actors not to focus on specific measures, but to address universal access to assistive technology from a holistic systems approach. This is in line with the conclusions MacLachlan and Scherer have put forward in their work on systems thinking for assistive technology. They contend that “without understanding and acting through the interconnections that pattern complex systems—such as those characteristic of assistive technology systems—our impacts will be necessarily partial, restricted and often marginalizing. A systems thinking approach allows for a meaningful linking of components and processes, a more realistic understanding of why and where initiatives might fail or succeed, and a more satisfying way of placing the user of assistive technology at the center of ideas, activities and outcomes” [28] (p.495).

The present study has revealed important evidence and provided direction for improving access to assistive technology based on comprehensive data analyses. In the past, and in line with the global priority research agenda for improving access to assistive technology, much of the research in this field has focused on assistive products, which has led to important advancements. However, the findings from this study underscore the immediate need to develop and strengthen research that addresses Provision but also Policy and Personnel, which were also included among the global priority research thematic areas of the agenda [29]. This study demonstrates the need for continuous research that collects and analyzes data from various countries to inform the effective development and provision of assistive products and services, as well as monitor the outcomes in the population and the system. Moreover, there is a need for studies of specific initiatives to better understand what role, if any, they can play in improving access to assistive technology.

### 4.2. Discussion of the Methods

Since the present study is observational, the results provide indications of the relative importance of the independent variables for predicting access, rather than the exact effects of the individual independent variables on the degree of access. To generate the latter type of information, an experimental design would have been required, which is hard to accomplish in this type of study. The results are still relevant as the analysis builds on the view that the independent variables exert a joint effect on the dependent variable, as a system of interrelated variables, rather than a collection of variables independent from each other. This view is highly relevant for this study as the studied variables are part of a real-world societal system, with both unique and joint influence on the societies’ ability to fulfill their citizens’ need for assistive technology.

The limitations of government and population surveys have been reported in previous studies [7,16]. This includes potential inconsistencies in survey translation, particularly in the population survey, and the standardized questions not capturing context-specific elements in the assistive technology system of a country. Due to data availability at the time of preparing this publication, the study included a relatively small sample of 20 countries. Despite these limitations, the countries included in this study exhibit a diverse range of health, education, and socioeconomic statuses, as measured by the Human Development Index (HDI) [30] (ranging from 0.54 to 0.95 among the included countries). As a result, the findings, particularly the correlations between assistive technology system elements and self-reported access among the population, are relevant and provide important insights to be generalized in a global context.

## 5. Conclusions

The study findings have highlighted that geographic coverage of assistive technology services in all functional domains is an instrumental system element to achieve universal access to assistive technology. However, it does not come without implementation of appropriate policies, sufficient funding, recruitment of adequately trained personnel and availability of the assistive products a population needs. From the perspective of access at the national level, the value of specific initiatives remains unclear and calls for further studies.

## Figures and Tables

**Table 1:** Mapping of assistive technology system elements to the components of the 5P model.

System Element	People	Products	Provision	Personnel	Policy
Sectors with legislation					X
Functional domains covered by legislation					X
Sectors with budgets					X
Responsible ministries					X
Districts with services related to [functional domain]			X		
Functional domains with adequate personnel				X	
Functional domains with training				X	
Financial measures					X
Regulatory measures		(X) ^1^	(X) ^1^		X
Specific initiatives	X	X	X		X

^1^ Some regulatory measures concern assistive products and provision, i.e., policies within the Products and Provision components of the 5P model.

**Table 2:** Survey characteristics and access to assistive technology.

Country (Region)	Sample Size	Interview Methods *	Access Including Spectacles (%)	Access Excluding Spectacles (%)
Azerbaijan	5586	CAPI	35.4	16.8
Bhutan	11,930	CAPI	41.5	22.6
Djibouti	11,720	CAPI	14.7	7.5
Dominican Republic **	5003	CATI	52.8	48.0
Georgia	6864	CAPI	54.5	40.2
Guatemala (Solola)	2868	CAPI	15.7	9.1
Iran	18,870	CAPI	72.1	52.4
Iraq	14,220	CAPI	44.2	30.9
Italy	10,170	CAWI/CATI	87.1	77.2
Jordan	13,416	CAPI	70.3	55.5
Malawi (Blantyre)	9340	CAPI	10.5	7.6
Maldives	6843	CAPI	64.5	56.9
Myanmar	8743	CAPI	26.7	23.6
Nepal	11,230	CAPI	50.3	25.5
Pakistan	62,723	CAPI	22.3	9.7
Poland	6694	CAPI/CATI	88.3	81.4
Rwanda	7156	CAPI	9.3	8.2
Sweden **	1479	CATI	89.8	83.5
Tajikistan (Sughd)	2500	CAPI	48.2	18.3
Ukraine	7069	CAPI	79.6	60.9

* CAPI/CATI/CAWI = Computer Assisted Personal/Telephone/Web Interviewing; ** 18 years and older.

**Table 3:** Descriptive statistics of independent and dependent variables (*N* = 20).

Variables	Countries with Data (*n*)	Min	Max	Mean	Standard Deviation
Access including spectacles	20	9.3%	89.8%	48.9%	26.2%
Access excluding spectacles	20	7.5%	83.5%	36.8%	25.3%
Districts with services related to:					
Cognition	11	0%	100%	65.7%	45.5%
Communication	12	0%	100%	70.4%	43.3%
Hearing	13	0%	100%	72.0%	38.7%
Mobility	15	13.0%	100%	75.9%	35.4%
Self-care	12	0%	100%	70.9%	42.7%
Vision	14	16.7%	100%	88.2%	23.7%
				**Median**	
Sectors with legislation	19	1	7	4	
Functional domains covered by legislation	19	1	6	6	
Sectors with budgets	18	0	7	2	
Responsible ministries	20	1	6	3	
Functional domains with adequate personnel	16	0	6	1	
Functional domains with training	19	0	6	3	
Financial measures	20	0	5	2	
Regulatory measures	19	0	7	4	
Specific initiatives	20	1	8	5	

**Table 4:** Statistics of OPLS models predicting access including and excluding spectacles, respectively. *R*^2^*X* and *R*^2^*Y* are the fractions of the variation in the independent and dependent variables, respectively, that are explained by the predictive component in the model. *Q^2^Y* is a measure of the model’s ability to predict omitted Y data. All three statistics range from 0 to 1 (representing perfect fit or predictive ability). *Comp* refers to the number of significant predictive components in the model and the number of components capturing any systematic variation in the X and Y data, respectively, that remains after the predictive component was extracted (i.e., orthogonal variation) (P + OX + OY).

Model	*Comp*	*R* ^2^ *X*	*R* ^2^ *Y*	*Q* ^2^ *Y*
Access including spectacles	1 + 0 + 0	0.34	0.59	0.49
Access excluding spectacles	1 + 0 + 0	0.35	0.51	0.39

**Table 5:** Assistive technology system elements sorted according to the magnitude of association with access to assistive products including spectacles.

System Elements	5P Components
Districts with services *	Provision
Regulatory measures *^,1^ Sectors with budgets * Functional domains covered by legislation Responsible ministries ^2^ Financial measures ^2^ Sectors with legislation ^2^	Policy
Functional domains with adequate personnel	Personnel
Functional domains with training	
Specific initiatives	People, Products, Provision, Policy

* Statistically significant associations with access to assistive products; ^1^ Some of the regulatory measures concern assistive products and provision. However, in this table they have been categorized under Policy; ^2^ The order of these three system elements differed between access including and excluding spectacles, respectively. However, the differences in the non-significant magnitudes of associations were small, see Figure 1 and Figure 2.

## Data Availability

Data analyzed in this study are available through WHO Global Health Observatory Assistive Technology Data Portal. https://www.who.int/data/gho/data/themes/assistivetech (accessed on 28 April 2023). Data on population met need for assistive technology in Bhutan and Rwanda were not published in the portal at the time of submitting this manuscript.

## Supplementary Materials

The following supporting information can be downloaded at: https://www.mdpi.com/article/10.3390/healthcare11091313/s1, Table S1. Assistive technology system elements. Empty cells indicate missing data.; Table S2. Decomposed assistive technology system element measures.
