# Supplementary material for: On the Relation between Assistive Technology System Elements and Access to Assistive Products Based on 20 Country Surveys
Source: Healthcare (Basel). 2023 May 3;11(9):1313. doi: 10.3390/healthcare11091313 (PMC10178385; doi:10.3390/healthcare11091313)
Supplement: Supplementary file 1 [file healthcare-11-01313-s001.zip › healthcare-2314583-supplementary.pdf]

Supplementary Materials

Table S1. Assistive technology system elements. Empty cells indicate missing data.

Country	Number of government sectors with assistive technology-related legislation	Number of functional domains covered by assistive technology-related legislation	Number of government sectors with budgets that cover assistive technology	Number of ministries responsible for assistive technology	Proportion of districts (or similar) with services related to cognition (%)	Proportion of districts (or similar) with services related to communication (%)	Proportion of districts (or similar) with services related to hearing (%)	Proportion of districts (or similar) with services related to mobility (%)	Proportion of districts (or similar) with services related to self-care (%)	Proportion of districts (or similar) with services related to vision (%)	Number of functional domains with adequate human resources for assistive technology	Number of functional domains for which training on assistive technology are available	Number of financial measures related to assistive technology	Number of regulatory measures related to assistive technology	Number of specific assistive technology initiatives
Azerbaijan	6	6	6	4							6	6	2	4	8
Bhutan	1	6	1	1	100	100	100	100	100	100	3	3	1	2	2
Djibouti			1	3	16.7	16.7	16.7	16.7	16.7	16.7			1		3
Dominican Republic	7	6	7	5	100	100	100	100	100	100	2	3	5	1	1
Georgia	7	4	3	5	100	100	100	100	100	100	2	0	1	4	7
Guatemala	6	4	1	2								3	2	3	6
Iran	6	6	7	6	100	100	100	100	100	100	1	3	2	4	5
Iraq	1	2		2				100			2	2	1	4	6
Italy	4	6	1	4								3	3	3	1
Jordan	6	6		3							0	5	4	3	1
Malawi	6	6	0	4	0	0	6.9	13.8	0	96.6	1	1	0	0	5
Maldives	3	6	2	4	100	100	100	100	100	100	0	0	2	7	3
Myanmar	5	6	5	4				35.3	100	100	0	4	1	4	7
Nepal	2	6	2	2			61.0	13.0		54.6	0	5	2	4	6
Pakistan	2	6	3	3	6.2	6.2	31.2	43.8	12.5	62.5	3	3	3	1	6
Poland	3	4	3	3		100	100	100		100		6	3	4	2
Rwanda	1	1	2	2	0	0	0	100	0	100	0	4	2	1	5
Sweden	4	6	3	3	100	100	100	100	100	100	6	6	2	7	8
Tajikistan	2	6	2	2	100	100	100	100	100	100	0	0	1	3	2
Ukraine	3	6	2	4		92.6	92.6	92.6	92.6	92.6	0	3	1	4	8

Table S2. Decomposed assistive technology system element measures.

Assistive technology system element	n (%)
Legislation on assistive technology	
In separate legislation	6 (33)
In legislation on health services (or similar)	15 (83)
In legislation on social services (or similar)	15 (83)
In legislation on education (or similar)	11 (69)
In legislation on labour (or similar)	11 (65)
In legislation on defence (or similar)	8 (53)
In other legislation(s)	9 (53)
People covered by legislation: those with difficulties in...	
Cognition	15 (88)
Communication	17 (94)
Hearing	18 (95)
Mobility	18 (95)
Self-care	14 (82)
Vision	17 (94)
Budget for assistive technology	
In separate budget	6 (35)
In budget for health services (or similar)	13 (76)
In budget for social services (or similar)	11 (79)
In budget for education (or similar)	7 (58)
In budget for labour (or similar)	5 (42)
In budget for defence (or similar)	5 (42)
In other budget(s)	6 (60)
Responsible ministries	
Health (or similar)	20 (100)
Social (or similar)	16 (80)
Education (or similar)	9 (56)
Labour (or similar)	8 (50)
Defence (or similar)	6 (43)
Other(s)	7 (58)
Adequate human resources in the domain of...	
Cognition	2 (13)
Communication	2 (14)
Hearing	6 (38)
Mobility	8 (50)
Self-care	2 (13)
Vision	6 (43)
Training on assistive technology in the domain of...	
Cognition	4 (25)
Communication	6 (38)
Hearing	13 (72)
Mobility	15 (79)
Self-care	7 (41)
Vision	15 (83)
Financial measures	
Public insurance schemes	13 (65)
Compulsory private insurance schemes	5 (38)
List of subsidized or free assistive products	12 (71)

Other(s)	6 (55)
Regulatory measures	
Safety of assistive products	9 (53)
Procurement of assistive products	14 (78)
Delivery of assistive technology services	11 (65)
Qualifications of assistive product providers	9 (56)
Accessible environments	12 (71)
Assistive products in emergency preparedness or response programmes	4 (31)
Accessible environments in emergency preparedness or response programmes	4 (40)
Specific assistive technology initiatives	
Affordability of assistive products	12 (71)
Development of assistive products	9 (56)
Procurement of assistive products	14 (82)
Service delivery capacity	14 (82)
Collection of data on population-based needs	15 (79)
Information to users and their families	13 (79)
Participation of assistive product users in planning and monitoring services	8 (72)
International collaboration on manufacturing, procurement or supply of assistive products	7 (54)
